# Trajectory of patients consulting the emergency department for high blood pressure values

**DOI:** 10.1007/s43678-022-00307-y

**Published:** 2022-05-03

**Authors:** Alain Vadeboncoeur, Marie-Joelle Marcil, Samuel Cyr, Mona Gupta, Alexis Cournoyer, Anthony Minichiello, Dominic Larose, Julie Sirois-Leclerc, Jean-Claude Tardif, Josée Morin, Violaine Masson, Mariève Cossette, Judith Brouillette

**Affiliations:** 1 Research Centre, Montréal Heart Institute, Université de Montréal, 5000 Belanger Street, Montréal, QC H1T 1C8 Canada; 2 Department of Psychiatry and Addiction, Faculty of Medicine, Université de Montréal, Montréal, QC Canada; 3 Faculty of Pharmacy, Université de Montréal, Montréal, QC Canada; 4 Centre de recherche, Centre Hospitalier de L’Université de Montréal, Montréal, QC Canada; 5 Centre de recherche, Hôpital du Sacré-Cœur de Montréal, Université de Montréal, Montréal, QC Canada; 6 Centre de recherche, Hôpital Maisonneuve-Rosemont, Université de Montréal, Montréal, QC Canada; 7 Montreal Health Innovations Coordinating Center, Montréal, QC Canada

**Keywords:** Hypertension, Emergency, Patient-reported experience measures, Blood pressure

## Abstract

**Objectives:**

Emergency department (ED) visits for high blood pressure are increasing in frequency. We aimed to map those patients’ trajectory, from referral sources to the type of care received at the ED to anticipated actions for future high blood pressure concerns, and to better understand their reasons for consulting the ED for high blood pressure values.

**Methods:**

Between 2018 and 2020, patients who presented to the Montreal Heart Institute’s ED for elevated blood pressure were recruited in a prospective observational study including a post hoc structured telephone interview and medical chart review. Five possible referral sources were predetermined. We provided proportions and 95% confidence intervals.

**Results:**

A total of 100 patients were recruited (female: 59%, mean age: 69 ± 12). A majority (93%, 95% CI 88–98%) possessed a home blood pressure device, among which 46% (95% CI 36–56%) remembered receiving advice for its use. The main referral sources for high blood pressure to the ED were self-reference (53%, 95% CI 43–63%), advice of a lay person (19%, 95% CI 11–27%) or a nurse (13%, 95% CI 6–20%). Mainly, patients reported being concerned by concomitant symptoms or experiencing acute medical consequences (44%, 95% CI 34–54%), having followed the recommendation of a third party (33%, 95% CI 24–42%), or having concerns about their medication (6%, 95% CI 1–11%). Two weeks following their ED visits, consulting ED remained the main choice for future concerns about high blood pressure for 27% of participants. When specifically asked if they would return to the ED for elevated blood pressure, 73% (95% CI 64–83%) said yes.

**Conclusions:**

Most patients who consulted the ED for elevated blood pressure values were self-referred. More can be done to promote blood pressure education, effective use of personal blood pressure devices, and recommendations for patients and health professionals when confronted with high blood pressure results.

**Supplementary Information:**

The online version contains supplementary material available at 10.1007/s43678-022-00307-y.

## Résumé

**Objectifs:**

Les visites aux services d'urgence pour hypertension artérielle (TA) sont de plus en plus fréquentes. Nous avons cherché à cartographier le parcours de ces patients, depuis les sources d'orientation jusqu'au type de soins reçus aux urgences, en passant par les mesures prévues en cas de problèmes futurs de tension artérielle élevée, et à mieux comprendre les raisons pour lesquelles ils consultent les urgences pour des valeurs de tension artérielle élevées.

**Méthodes:**

Entre 2018 et 2020, les patients qui se sont présentés aux urgences de l'Institut de cardiologie de Montréal pour une TA élevée ont été recrutés dans le cadre d'une étude observationnelle prospective comprenant une entrevue téléphonique structurée post-hoc et un examen des dossiers médicaux. Cinq sources de référence possibles ont été prédéterminées. Nous avons fourni des proportions et des intervalles de confiance à 95 %.

**Résultats:**

Au total, 100 patients ont été recrutés (femmes : 59 %, âge moyen : 69 ± 12). Une majorité (93%, IC à 95% 88-98%) possédait un tensiomètre à domicile, parmi lesquels 46% (IC à 95% 36-56%) se souvenaient avoir reçu des conseils pour son utilisation. Les principales sources d'orientation vers les urgences en cas de tension artérielle élevée étaient l'auto-référence (53 %, IC 95 % 43-63 %), le conseil d'un tiers non-professionnel de la santé (19 %, IC à 95 % 11-27 %) ou d'une infirmière (13 %, IC à 95 % 6-20 %). Principalement, les patients ont déclaré être préoccupés par des symptômes concomitants ou des conséquences médicales aiguës (44 %, IC à 95 %, 34-54 %), avoir suivi la recommandation d’un tiers (33 %, IC à 95 %, 24-42 %) ou avoir des préoccupations au sujet de leurs médicaments (6 %, IC à 95 %, 1-11 %). Deux semaines après leur visite au service d'urgence, la consultation du service d'urgence est restée le principal choix en cas de préoccupations futures concernant l'hypertension artérielle pour 27 % des participants. À la question spécifique de savoir s'ils retourneraient aux urgences pour une TA élevée, 73% (IC à 95% 64-83%) ont répondu oui.

**Conclusions:**

La plupart des patients qui ont consulté les urgences pour des valeurs élevées de la tension artérielle se sont adressés d'eux-mêmes. Il y a place à l'amélioration pour promouvoir l’éducation sur la TA, l’utilisation efficace des appareils de pression artérielle personnels et les recommandations aux patients et aux professionnels de la santé lorsqu’ils sont confrontés à des résultats élevés en matière de TA.

## Clinician’s capsule

**Table:** 

***What is known about the topic?***
ED visits for high blood pressure are increasing.
***What did this study ask?***
What is the trajectory of patients seeking ED care for high blood pressure?
***What did this study find?***
The majority of participants were self-referred, used a personal blood pressure device and don’t recall receiving information about its use.
***Why does this study matter to clinicians?***
Improving high blood pressure patients’ education and professional advice should be prioritized to limit non-urgent ED visits.

## Introduction

Hypertension is managed by primary healthcare providers. While literature does not support the emergency department (ED) as the optimal place for evaluation and treatment of elevated blood pressure [1, 2], ED visits for hypertension increased by 64% from 2002 to 2012 in Ontario [3]. The trajectory of patients who consult the ED for a chief complaint of high blood pressure is not well known. Some patients are referred by healthcare professionals to rule out acute target-organ damage, defined as hypertensive emergency, which has a prevalence of 2/1000 ED visits [4].

Many people can measure their blood pressure using a home or pharmacy device. Clinical benefits include blood pressure measurement under less stressful circumstances (“non-white coat”) and optimizing antihypertensive adjustments [5]. Nonetheless, individuals may encounter abnormal blood pressure readings without knowing how to respond. Recent directives published for patients regarding home blood pressure monitoring state that elevated readings “*warrant a timely appointment with a primary care provider*” and that “*patients with elevated readings who are experiencing symptoms of a heart attack or stroke should seek immediate medical assessment.*” [6]. Awareness of the referral sources and the reasons patients visit ED for elevated blood pressure could serve as essential background knowledge for initiatives targeting the development of care pathways for community-dwelling patients with elevated blood pressure.

This study’s objective was to describe the referral sources and reasons of patients visiting the ED for elevated blood pressure. We also verified ownership of a home blood pressure device and the ED care received.

## Methods

This prospective observational cohort study was conducted from October 2018 to January 2020 at the Montreal Heart Institute (MHI)’s ED. The MHI’s ED is an open-access ED mainly devoted to cardiac emergencies with an annual census of 18,000 patients. The project was approved by the MHI’s Research and Development Ethics Board.

The target population was adults presenting to the ED with elevated blood pressure values as their chief complaint, as evaluated by the Canadian Triage and Acuity Scale (CTAS). An ED nurse or physician asked those patients if they agreed to be informed about this study while being evaluated in the ED. Then the research team called patients who accepted within two weeks following their ED visit to obtain consent to participate. After obtaining the consent, we conducted a structured interview (see Appendix A). Two team members independently categorized the respondents’ answers according to predetermined categories. Participants’ medical records were reviewed to gather sociodemographic and medical information, blood pressure values at the ED triage and discharge, healthcare services received, and presence of hypertensive emergency diagnosis.

This study’s main objective was to identify referral sources of patients presenting to the ED for elevated blood pressure values. Five referral sources were predetermined: (1) self-referral; (2) lay person; (3) pharmacist; (4) nurse; or (5) physician. We also described participants’ main reasons for consulting the ED for high blood pressure, the proportion of ownership of home blood pressure devices and the type of care received.

Descriptive statistics were reported for continuous variables, while frequencies and percentages were presented for categorical variables. The 95% exact or Wald confidence intervals (CI) were provided for proportions. Assuming an expected proportion up to 50% of one of the five referral sources, a sample size of 100 patients would allow the precision of ± 10% for the proportion using a two-sided 95% CI. Analyses were conducted using SAS software, version 9.4 (SAS Institute Inc., Cary, NC, USA).

## Results

The study included 100 patients (female: 59%, mean age: 69 ± 12) (See Appendix B for participants’ inclusion flowchart and C for patients’ characteristics). A majority (93%, 95% CI 88–98%) possessed a home blood pressure device, among which 46% (95% CI 36–56%) remembered receiving advice for its use. When asked ‘Do you know what an abnormal blood pressure value is?’, 89% (95% CI 82–96%) answered positively, reporting median blood pressure values of 140/90 as abnormal. Almost all patients (90%, 95% CI 84–96%) stated having a family physician, with 61% (95% CI 50–72%) reporting having access if they had a new health concern.

Table 1 describes the participants’ referral sources. The majority of participants were self-referred (53%, 95% CI 43–63%). Of the participants referred by nurses, most (62%) received this advice through a non-urgent healthcare telephone line (811). The main reasons patients reported were being concerned by concomitant symptoms (e.g., headache, chest pain, pounding heart, dizziness) or fearing developing acute medical consequences (e.g., stroke, myocardial infarction) (44%, 95% CI 34–54%), having followed the recommendation of a third party (33%, 95% CI 24–42%), or being concerned about medication (6%, 95% CI 1–11%).

**Table 1:** Frequency, binomial proportion and 95% confidence intervals for the referral sources leading patients to the emergency department for high blood pressure (*n* = 100)

Referral sources	Frequency	Proportion (%)	95% CI	
			LCI (%)	UCI (%)
Self-reference	53	53.0	43.2	62.8
Lay person	19	19.0	11.3	26.7
Nurse	13	13.0	6.4	19.6
Pharmacist	9	9.0	3.4	14.6
Physician	5	5.0	0.7	9.3
Other healthcare professional	1	1.0	0.0	5.5

Data are presented as frequency (*n*) and binomial proportion (%)

*CI* confidence interval, *LCI* lower confidence intervals, *UCI* upper confidence interval

All patients received a routine electrocardiogram (ECG), a procedure done on every patient seen at the MHI’s ED for general complaints, 47% (95% CI 37–57%) received blood tests, and 5% (95% CI 1–9%) underwent further medical imaging procedures. Eight patients (8%, 95% CI 3–13%) received oral antihypertensive medication, and none required intravenous treatment. Nearly 1 in 4 participants (23%, 95% CI 15–31%) had their current prescription adjusted or received an additional prescription for antihypertensive medication. One (1%, 95% CI 0–6%) was admitted to the short stay unit. No patients received a hypertensive emergency diagnosis, and none of them were admitted to the ward. See Appendix D for the ED care received.

When asked, ‘If you have an elevated blood pressure measurement again, what are you going to do?’ the main answers were: returning to the ED (27%, 95% CI 17–36%), consulting a family doctor/clinic (24%, 95% CI 16–33%), and using relaxation/stress management strategies (19%, 95% CI 11–27%). When specifically asked if they would return to the ED for elevated blood pressure, 73% (95% CI 64–83%) said yes.

## Discussion

Although a large Canadian study [6] explored the trajectory of patients with a final diagnosis of hypertension, our study is the first, to our knowledge, to explore the trajectory of patients who consult the ED for a chief complaint of high blood pressure. The main referral sources for patients presenting in the ED for elevated blood pressure were themselves, followed by the recommendations of a lay person (e.g., friends, families). Nurses were the most frequent referral sources among healthcare professionals, which was driven by telehealth nurses. These references may have followed guidelines recommendations stating high blood pressure accompanied by non-specific symptoms, such as headache and chest pain, warrants further investigation [7]. Although most patients reported having a home blood pressure monitor, more than half reported not having received instruction on how to use it.

The fear of experiencing an acute medical condition and the interpretation of concurrent symptoms as significant drove participants to visit the ED for their high blood pressure values. Third parties played a major role, inciting a third of these visits. This finding is consistent with other work showing that non-healthcare professionals, particularly family members, play an important role in patients’ decision-making process [8]. Also, we found that re-consulting the ED if elevated blood pressure reoccurs remains participants’ top action. This finding may also reflect the patient’s perception of need and urgency surrounding high blood pressure, which has been documented to drive non-urgent ED visits [9]. Few patients studied underwent investigations, except for routine ECG, probably in relation to low acuity condition.

### Limitations

Results should be interpreted cautiously considering the following limits. Participants were contacted on average 14 days following their visit, which may have induced a recall bias. To be included in the study, patients were required to answer our calls during working hours. Results may not generalize to daytime workers and may underrepresent hospitalized/deceased patients. Information about the study and subsequent recruiting being at the discretion of the ED physician/nurse may have introduced a selection bias. Although we cannot be sure that all the patients with a main chief complaint of high blood pressure were proposed for the project, all patients who agreed were contacted by the research team, and the 100 patients included were those who were reached and accepted to be involved. Furthermore, we cannot provide data about those who refused to participate or were not reachable. Finally, because of our centre’s specificity, our results may not be generalizable to other centres.

### Implications

Both patients and their relatives should be targeted for blood pressure education. Guidelines recommend blood pressure self-monitoring [7, 10], implying that individuals must interpret their own readings, which may lead some to feel insecure. We found a disparity between ownership of a home blood pressure device and recall of advice from primary care providers on its use. There is an opportunity to provide blood pressure education and counseling, including the proper use of home blood pressure device, interpretation of readings, and what to do when confronted with elevated blood pressure reading. Future research could explore our results in general ED, assess how to improve patient education about when to present to the ED for elevated blood pressure, and find ways to harmonize information and professional guiding of patients, including telehealth nurses.

### Conclusion

Most patients who consulted the ED for elevated blood pressure values were self-referred and discharged. More can be done to promote blood pressure education, effective use of personal blood pressure devices and patient and health professional’s decisions when confronted with high blood pressure results.

## Supplementary Information

Below is the link to the electronic supplementary material.

Supplementary file1 (DOCX 77 KB)

## References

[1] Saito T, Hasebe N (2021). Malignant hypertension and multiorgan damage: mechanisms to be elucidated and countermeasures. Hypertens Res.

[2] Miller J, McNaughton C, Joyce K, Binz S, Levy P. Hypertension management in Emergency Departments. Am J Hypertens. 2020. 10.1093/ajh/hpaa068 PMC7577644 32307541

[3] Masood S, Austin PC, Atzema CL. A population-based analysis of outcomes in patients with a primary diagnosis of hypertension in the Emergency Department. Ann Emerg Med. 2016;68(3):258–67 e5. 10.1016/j.annemergmed.2016.04.060 27395439

[4] Janke AT, McNaughton CD, Brody AM, Welch RD, Levy PD. Trends in the incidence of hypertensive emergencies in US Emergency Departments from 2006 to 2013. J Am Heart Assoc. 2016;5(12). 10.1161/JAHA.116.004511 PMC5210448 27919932

[5] Shimbo D, Artinian NT, Basile JN, Krakoff LR, Margolis KL, Rakotz MK, et al. Self-measured blood pressure monitoring at home: a joint policy statement from the American Heart Association and American Medical Association. Circulation. 2020;142(4):e42–63. 10.1161/CIR.0000000000000803 32567342

[6] Mostarac I, Thomas J, Atzema C (2021). Mesure de la tension artérielle à domicile: directives à l’intention des patients canadiens. Can Med Assoc J.

[7] Unger T, Borghi C, Charchar F, Khan NA, Poulter NR, Prabhakaran D (2020). 2020 International society of hypertension global hypertension practice guidelines. Hypertension.

[8] Lamore K, Montalescot L, Untas A (2017). Treatment decision-making in chronic diseases: What are the family members’ roles, needs and attitudes? A systematic review. Patient Educ Couns.

[9] Uscher-Pines L, Pines J, Kellermann A, Gillen E, Mehrotra A (2013). Emergency department visits for nonurgent conditions: systematic literature review. Am J Manag Care.

[10] Rabi DM, McBrien KA, Sapir-Pichhadze R, Nakhla M, Ahmed SB, Dumanski SM (2020). Hypertension Canada’s 2020 comprehensive guidelines for the prevention, diagnosis, risk assessment, and treatment of hypertension in adults and children. Can J Cardiol.

