# Supplementary material for: Trajectory of patients consulting the emergency department for high blood pressure values
Source: CJEM. 2022 May 3;24(5):515–9. doi: 10.1007/s43678-022-00307-y (PMC9345802; doi:10.1007/s43678-022-00307-y)
Supplement: Supplementary file 1 — Supplementary file1 (DOCX 77 KB) [file 43678_2022_307_MOESM1_ESM.docx]

**Appendix**

**Appendix A**

**Interview questions/ protocol**

1. **Why did you come to the Montreal Heart Institute’s emergency room for high blood pressure?**
   1. What could have happened if you had not come to the emergency room?
2. **Did anyone advise you to come to the emergency room for high blood pressure?**

*If a person has advised:*

- 1. Who is this person?

*If not a health professional:*

- - 1. Why did this person think you needed to come to the emergency room?
    2. (*According to the previous answer*) She was worried/afraid of what?

*If a health professional:*

1. What is his profession?
2. Where does she work?
3. Why did this person think you needed to come to the emergency room?
4. (*According to the previous answer*) She was worried/afraid of what?
5. **In the emergency room, what have they done for your high blood pressure?**
6. Have you followed the recommendations?
7. Why?
8. **In the emergency room, what have you been told/recommended for your high blood pressure?**
9. Have you followed the recommendations?
10. Why?
11. **If you have high blood pressure again,**
    1. **What are you going to do?** (*Open-ended question)*
    2. **Are you going to come to the emergency room?** *(Closed-ended question)*
12. Why?
13. **Do you have a device that measures blood pressure at home?**
14. Did anyone tell you how the device works?

*If no:*

1. How did you find out how it works?

*If yes:*

1. Who?
2. Do you know what an abnormal BP value is?

*If yes:*

1. What are the abnormal blood pressure values?
2. Who explained to you those values?

*If no:*

1. How did you find out what were the abnormal values?
2. Has anyone told you what to do when the blood pressure values are abnormal?
3. Who?
4. What to do?
5. **Do you have a family doctor?**

Do you have access to your doctor if you have a new health concern?

**Appendix B**

Flowchart representing the inclusion of participants

Patients approached in the ED

**154** accepted to be contacted

4 excluded: language barrier

6 declined to participate

44 not included:

- 39 not reached
- 4 not eligible for inclusion: no high BP as main complain
- 1 death

**110** reached

**100** included

**Appendix C**

Sociodemographic and medical characteristics of patients (*n* = 100)

| **Variables** | **Patients (*n* = 100)** |
| --- | --- |
| **Sex** |  |
| Female | 59 (59) |
| Male | 41 (41) |
| **Mean age** (*SD*), years | 69 (12) |
| **Ethnic group** |  |
| White | 79 (79) |
| Black | 1 (1) |
| Hispanic | 4 (4) |
| Other | 16 (16) |
| **Marital status** |  |
| Married or in couple | 51 (51) |
| Divorced | 18 (18) |
| Single | 15 (15) |
| Widowed | 16 (16) |
| **Medical history** |  |
| Hypertension | 73 (73) |
| Heart disease | 41 (41) |
| Family history of heart disease | 30 (30) |
| Dyslipidemia | 49 (49) |
| Diabetes | 11 (11) |
| Stroke | 4 (4) |
| Family history of stroke | 2 (2) |
| Kidney failure | 1 (1) |
| Pulmonary edema | 0 (0) |
| Psychiatric disorder | 18 (18) |
| Chronic conditions | 40 (40) |
| neurological* | 1 (1) |
| musculoskeletal* | 6 (6) |
| pulmonary* | 9 (9) |
| digestive* | 18 (18) |
| endocrine* | 15 (15) |
| other * | 1 (1) |
| Cancer | 12 (12) |
| **Medication** |  |
| Antihypertensives (*n* = 92) | 68 (74) |
| **Do you have a family doctor?**, yes (*n* = 100) | 90 (90) |
| Do you have access to your doctor if you have a new health concern?, yes (*n* = 74) | 45 (61) |
| **Do you have a device that measures BP at home?**, yes (*n* = 100) | 93 (93) |
| Did anyone tell you how the device works?, yes (*n* = 93) | 43 (46) |
| **Do you know what an abnormal BP value is?**, yes (*n* = 80) | 71 (89) |
| **Who explained abnormal BP values** (*n* = 63) |  |
| Health Professional | 24 (38) |
| Patient’s personal knowledge | 20 (32) |
| Information displayed at the pharmacy | 7 (11) |
| Booklet that comes with BP home device | 6 (10) |
| Friends and family | 3 (5) |
| Media | 3 (5) |

* Categories of chronic conditions are not mutually exclusive.

*Notes:* Data are presented as numbers (%) or as mean (*SD*).

Abbreviation: BP: blood pressure, SD: standard deviation.

**Appendix D**

Type of care received at the emergency department

| **Emergency care** | **Proportion** | **95 CI** | |
| --- | --- | --- | --- |
|  |  | **LCI** | **UCI** |
| **Medical laboratory** | 47.0% | 37.2% | 56.8% |
| **Medical imaging** | |  |  |
| ECG | 100.0% |  |  |
| Other medical imaging (echography, scan or radiography) | 5.0% | 0.7% | 9.3% |
| **Specialist consultation** | 1.0% | 0.0% | 5.5% |
| **Medication** |  |  |  |
| Given at the ED | 8.0% | 2.7% | 13.3% |
| Addition or adjustment of current prescription | 23.0% | 14.8% | 31.3% |
| **Patient education** | 95.0% | 90.7% | 99.3% |
| **Family physician follow up** | 66.7% | 57.4% | 76.0% |
| **Referral** |  |  |  |
| Outpatient | 22.0% | 13.9% | 30.1% |
| Community | 4.0% | 0.2% | 7.8% |
| **On ED bed** | 5.0% | 0.7% | 9.3% |
| **Short stay unit admission** | 1.0% | 0.0% | 5.5% |

*Notes:* Data are presented as binomial proportions (%).

Abbreviations: ED: emergency department, ECG: electrocardiogram, CI: Confidence interval, LCI: Lower confidence intervals, UCI: Upper confidence interval.
